# Massachusetts' General Hospital

**Published:** 1830-04-01

**Authors:** 


### XIII.

### MASSACHUSETTS' GENERAL HOSPITAL.

### I. REMOVAL OF HALF THE LOWER JAW BONE, FOR OSTEO-SARCOMA. By DR. WARREN.

The following case is taken from the 6th No. of the Boston Medical and Surgical Journal.

JAMES COOK, blockmaker, aged 17. Sixteen months since received a violent blow on the left side of the lower jaw. In four months after, he perceived an enlargement of the bone on that side, which for ten months was slow, but for the last month has been rapid. The tumor now produces a most unsightly projection of the face on the left side. At first view it appears to be seated on the front of the bone, but on examination is ascertained to extend inward to the mouth as well as outward. The whole of the left side of the jaw is found to be in an enlarged state from the chin to the ear; and the projection in front of the bone is of the size of a large egg. The Consulting Physicians of the hospital having examined the patient, agreed that nothing could save the patient's life but an operation. This was accordingly performed by Dr. WARREN on the following day.

*Operation. February*, 1825.—An incision was begun close to the fore part of the ear and carried downward to the angle of the jaw, thence forward, in a semicircular form to the chin, pursuing the line of the lower edge of the jaw. The skin and muscles were dissected upwards so as to uncover the jaw and the tumour. The dissection was then carried under and within the jaw, to separate it from the mouth. Next, the two front teeth of the left side were removed. Then the bone was sawn through at the chin by Hey's saw; in doing which, it was necessary to have the bone supported by an assistant, on account of its unsteadiness under the saw. In this way the bone being divided, the anterior part of it was drawn forwards and the knife carried on its inside, to complete the division of the flesh within, up to the joint. The bone being found to be enlarged quite into the socket, it was necessary to disarticulate it. In order to accomplish this the attachment of the temporal muscle to the coronoid process was cut off, and with less difficulty than was expected. After this, the ligaments of the joint being cut, the bone was removed from its socket.

The facial artery was divided; and the internal maxillary artery, being in contact with the tumor, was necessarily cut off. Both bled furiously, but were readily stopped by compressing the carotid artery firmly, while they were secured by ligature. The patient did not lose much blood; nor did he complain very much of the operation.

The skin was brought together and secured by plasters; and the large excavation, formed by taking out the bone, was filled by sponges on the outside of the dressings and well secured by bandage.

On the evening of the operation, the patient, after appearing well, was excessively faint; not from loss of blood but from constitutional sympathy; and great fears were felt for his life. By the use of cordials and external warmth he revived, and had no further bad symptoms afterwards. Great part of the wound was found united on the first dressing, and the rest healed well, so that in a month he was well enough to quit the hospital.

Notwithstanding the loss of half the lower jaw, he was able to bite and chew very comfortably. Before he left the hospital, some one asking him if he could use his jaw, he answered, "Give me a cracker (biscuit) and I will show you."

*Remarks*. Considering the importance of the parts involved in this operation, it is remarkable that it should be accomplished with so much ease, and so little ill consequence. The affection of the constitution at a period a few hours subsequent to the operation was indeed great, but soon passed off. Young persons suffer more by constitutional sympathy from operations, than old ones; that is, they are in more danger of being depressed and dying than old people, under the same loss of blood. Infants operated on for hare-lip, sometimes die from this cause, to the great astonishment of the surgeon. Children subjected to amputation and other considerable operations, now and then sink where there has been very little effusion of blood. Such occurrences should lead us to watch these young patients with more than ordinary attention. As an exemplification of the difference of impression made by operations on patients of different ages, compare the effect of this operation on a healthy muscular young man, with that related in the second number of this journal on a person of feeble constitution and advanced period of life.

### II. Excision of Nerves in Cases of Neuralgia. By J. C. Warren, M.D.

From a number of cases of this dreadful disease, reported from the above hospital in the Boston Medical and Surgical Journal, we select the three following as the most interesting. Their authenticity is unquestionable.

#### Case 1.—*Excision of the Submaxillary Nerve.*

*Mr. S.* aged 70, applied to me, stating that he had a dreadfully painful complaint in the face, and that he wished an operation for its relief. It appeared that he had been first affected without any obvious cause about fourteen years before. His pains were of two kinds; first, a constant aching pain, which he compared to the worst toothache; second, a spasmodic affection which occurred many times during the day. When he had the latter kind of attack, the muscles of the face quivered, the face became red and swelled, the eyes were filled with tears, and his intellect was for the moment suspended. For the last four years he had not been free from pain while awake, and his sleep was short and interrupted.

Respectable practitioners in the part of the country where he lived, had performed three operations, two on the sub-orbitar nerve and its branches, the third on the nerve of the lower jaw, where it comes out on the chin. These operations gave him a degree of relief, but the pain continued with a severity intolerable, and life had become a burden to him.

The pain he described as beginning near the ear, and thence darting into the lower and upper jaw, the lips, eye, forehead and scalp. The patient had made himself acquainted with the situation of the nerves of the face, and believed his pain to reside in the facial nerve, which he wished to have divided.

He was informed that an operation such as he wished might be executed; but that in his case the affection appeared so general that there was no prospect of a cure; and that, in fact, there were not any cases on record, of a successful division of the facial nerve at its root for this disorder.* As soon as the patient understood that the operation was practicable he desired to have it performed, and agreed to enter the Hospital, on account of the superior advantages it afforded over any private situation.

The operation was thus conducted. An incision two inches long was carried from the back of the ear downwards in front of the mastoid process. The edge of the parotid gland was then exposed on one side, and on the other, the anterior edge of the mastoid muscle. The dissection being continued between the parotid gland and the mastoid process, the facial nerve was exposed where it crosses this space and passes on the gland, it was divided and a portion cut out. When the nerve was cut, the muscles of the face quivered and were paralyzed; but the patient said he merely perceived the division; that it was not attended with an acute pain, and that the principal cause of his sufferings was not reached.

After this operation, it appeared that the pain in the upper part of the face was diminished; perhaps removed. But the patient now became sensible that the most acute pain, and that which probably had existed first, was seated deeply in the lower jaw, beginning at the zygomatic arch and shooting into the bone. It was entirely independent of the wound made in the operation. From this wound he experienced no inconvenience, was unwilling to speak of it, and scarcely wished it dressed, so greatly was he disappointed at not being relieved from his sufferings. He begged his case might be again taken into consideration, and something more if possible devised for his relief. A meeting of the consulting physicians and surgeons being called, I proposed the operation which is described hereafter, and it being agreed to, was performed nine days after the first.

An incision was made over the side of the jaw, from the semilunar notch to the inferior edge of the bone. The parotid gland being exposed, was divided as far back as possible, and turned

* The branches of the facial nerve have been repeatedly cut in front of the parotid gland; but we are not aware that the nerve has been divided at its root. At least Mr. Swan, in his prize book, lately published, informs us that "to attempt to divide the trunk of this nerve will not only be very difficult, but it will likewise be very dangerous." It is presumed he would have stated any instance of its division which he might have known. The second operation mentioned in this article is, we suppose, entitled to the merit of novelty.—Ed.

forwards. Then the masseter muscle was divided in the course of its fibres to the bone, and afterwards the edge of the knife being turned forwards, some of the fibres were transversely cut, in order to make room over the bone. A trephine, three quarters of an inch in diameter, was then applied half an inch below the semilunar notch, midway between the anterior and posterior edges of the jaw; and the circular piece sawed through and removed in two parts, the external table by a lever, the internal by forceps. Between these pieces lay the nerve, with its accompanying artery and vein, at the point where they penetrate the bone. At the superior edge of the hole in the bone was seen the large internal maxillary vein, pulsating from the movements of the artery. The sub-maxillary nerve being now raised on a probe, the patient directly exclaimed that this was the seat of his sufferings. Half an inch of this nerve was cut out, and on examination, it was found to comprehend the branch given to the internal face of the lower jaw. The artery was tied without difficulty. The transverse artery of the face had been previously tied on each side of the wound. A suture was employed to bring together the two parts of the parotid gland, and the wound closed by adhesive plaster.

The patient said that the pain of this operation could not be compared with that from his disorder. The pain on the wounding and dividing the maxillary nerve was most acute.

On the evening of the operation he was relieved from pain, the first time for four years; and had no return of it afterward. On passing a probe into the wound three or four days after, a branch of nerve in the masseter was touched, and a violent pain produced. The pain did not, however, return, nor had he any threatening of his former attacks. On the nineteenth day from the second operation, his wounds being nearly healed, he left the Hospital perfectly cured of his disease, with the strongest conviction it would not recur, and not a little gratified by his own perseverance.

Case 2.—*Excision of the Infra-Orbitar Nerve.*

Encouraged by the result of the last case, I operated in the same manner in the following cases.

*Mary Tucker, aged fifty-one, admitted into the Massachusetts General Hospital 24th April,* 1824.

Has a pain in the left side of the face, situated in the infra-orbitary foramen, but often extending to the jaws and occasioning a sense of tightness and burning in these parts. She describes the pain as acute and lancinating, and as always aggravated by exposure to cold and by fatigue. During the paroxysms her vision is much impaired, and when they are severe, the face swells very much. There are moments when she is free from complaint, but talking or exertion is apt to bring it on again. The disease is most troublesome at night, and prevents sleep. Says she has not slept all night for a number of years.

She has had this disease for eighteen years. For six months past has had almost constant pain. Says she has had the cow-pock, was very ill, and in her recovery had pain and stiffness in the left side of her face, with an inclination of the face to the left side. This lasted three months; during the warm weather she was free from this affection, but, in cold weather, it recurred, and has gradually assumed its present form. She had rather a feeble constitution originally, but enjoyed tolerable health previous to the present disease. Has occasionally used anodynes with advantage at night. Has always been rather costive.

℞. Solut. Sulph. Magnes. ℥iij.

29th.—*Operation.* The head was inclined to the right shoulder, resting upon the breast of an assistant. An incision was then made directly over the nerve, from the orbit, downwards about an inch. The seat of the nerve was made certain by passing a probe into the wound. Then the nerve being fully exposed, was divided near the infra-orbitar foramen; a piece half an inch long was dissected out and removed with its incipient ramifications. After the operation, a few small vessels bled considerably, which were restrained by sponges and cold water. The patient was then returned to her bed.

30th. At two o'clock, yesterday, the sponge was removed, and the vessels bled as copiously as before. A layer of lint was placed in the wound. The patient complained of some soreness and pain last evening, and was directed to take thirty drops of laudanum. This morning is tolerably comfortable; says she passed a quiet night. Now the situation of the pain has altered; since the operation, it is more confined to the parts about the lower jaw. Bowels well. Face a little swollen. From this time the pain gradually diminished, and in June she left the Hospital, entirely cured.

Case 3.—*Margaret Andrews, widow, aged 57. Entered the Mass. Gen. Hospital, March* 16, 1827.

Six years ago had a carious tooth in upper jaw, on left side; at the same time began to have darting or jumping pain on left side, commencing at this tooth and passing up the cheek. Had the tooth extracted with some relief. Paroxysms of pain have gradually increased in violence and duration—are worst, as she thinks, in August and September. They now commence as at first. The pain passes to the cheek-bone where it is most severe: it then starts in two courses, one passing the external, the other the internal angle of the left eye, and is again felt at a spot on the forehead. The pains sometimes extend to the back of the head. When the pain is most severe, she cannot bear the slightest touch upon the cheek. Severe paroxysms produce nausea; they are brought on by taking hot or cold substances, or by exposure to cold air. Her appetite is tolerably good; tongue clean,—is unable to extend it equally on both sides. Bowels prone to costiveness. Ceased menstruating at forty-eight or forty-nine; does not know which. Has used pills of extract of stramonium and ipecac. with some relief.

March 17. *Take of the extract of stramonium sixty grains, and make into twelve pills.* Let her take them every four hours, beginning with one pill and adding one to each dose. Also when the pain is urgent at night let her take two grains of the extract of stramonium.

March 20. Now takes six pills three times a day. 25. Pains diminished. Is very dizzy for an hour after each dose of pills. 26. Pains were severe; dizziness less. 30. *Take of the carbonate of iron, sixty grains three times a day.* April 5. Dose of the carbonate increased to eighty grains three times a day 11. Pains on the whole undiminished during nearly a month, while she has been in the hospital. Referred to the care of the surgeon.

A blister two inches square was applied to the cheek and dressed daily with a plaster consisting of extract of belladonna ℥iij., resinous plaster, half an ounce. 16. *Take of extract of belladonna, one grain three times a day.* This quantity produced nausea and dizziness; but was continued to the 22d, when her pains were as severe as ever. Omit all the remedies and let her take two ounces of the purgative solution, as preparatory to an operation, after two days of rest from medicine.

April 24. *The operation was thus performed.*

An incision one inch and a half long was made from a point half an inch below the middle of the orbit towards the middle of the left half of the upper lip; the periosteum was exposed to view with the sub-orbitar nerve lying thereon and beginning to divide. The nerve, as it arises from the sub-orbitar foramen, was cut across, then the inferior part being raised and dissected up, for half an inch, was cut off. The patient expressed some pain on the touching and division of the nerve, and at the same time a satisfaction at feeling that the source of her suffering was reached.

After the operation the tics never appeared; but she had some pain, which rapidly diminished till the 14th of the following month, when she was discharged: and I had the satisfaction to hear from her some months after, that she had no pain subsequently to her quitting the hospital.

